# Unlocking the potential of Contract Research Organizations in Africa’s clinical trials ecosystem

**DOI:** 10.1017/cts.2025.10194

**Published:** 2025-11-12

**Authors:** Shiferaw Tesfaye Tilahun, Tsegahun Manyazewal, Immanuel Azaad Moonesar, Hee Soo Kim

**Affiliations:** 1 Ethiopian Public Health Institute, Addis Ababa, Ethiopia; 2 Center for Innovative Drug Development and Therapeutic Trials for Africa (CDT-Africa), College of Health Sciences, Addis Ababa University, Addis Ababa, Ethiopia; 3 Mohammed Bin Rashid School of Government, Dubai, United Arab Emirates; 4 International Vaccine Institute, Seoul, South Korea

**Keywords:** Contract Research Organizations, clinical trials, Africa, opportunities, challenges

## Abstract

Despite representing 18% of the world’s population and 20% of the disease burden, only an estimated 2% of global clinical trials include at least one study site in Africa. This underscores the critical need for continued research on how to overcome clinical trial challenges on the continent. In countries with established reputations for clinical trials, Contract Research Organizations (CROs) play a vital role, accounting for half of the research workforce and effectively managing clinical trials for pharmaceutical, biotechnology, and medical device companies. In contrast, the potential of CROs in Africa’s clinical trials ecosystem remains largely unexplored. This narrative review discusses the challenges, opportunities, best practices, emerging trends, and prospects of clinical trials in Africa. Major challenges in clinical trial implementation in Africa stem from gaps in financial and human resources, infrastructure, and regulatory systems, while opportunities are linked to Africa’s large population, genetic diversity, disease burden, lower operating costs, positive economic outlook, and growing interest from global health and research players. Emerging trends, such as the decentralization of clinical trials and conducting trials during public health emergencies, offer promising avenues for maintaining research continuity. Ultimately, the paper proposes a context-specific framework, aimed at maximizing the effectiveness of CROs in the continent’s clinical trials ecosystem.

## Introduction

A Contract Research Organization (CRO) is a company that provides clinical trial services for the pharmaceutical, biotechnology, and medical device industries [1]. As CROs perform only small pieces of a large puzzle [2], there are many different kinds of CROs, but in the medical field, typical services include data management, trial logistics management, biostatistics, medical writing, regulatory affairs, clinical trial planning, site selection and initiation, recruitment support, clinical monitoring, and project management [1]. The global CRO market was valued at USD 16.5 billion in 2007 [3]. The CRO market experienced an annual growth rate of 14%–16%, reaching USD 24 billion by 2010. In that year, CROs represented around half of the research workforce involved in drug and medical product development [4]. The CRO market was estimated to be worth USD 39.7 billion in 2021. It is anticipated to increase from USD 45.5 billion in 2022 to USD 115 billion by 2030, meaning that it will expand at a compound annual growth rate (CAGR) of 12% from 2022 to 2030 [5].

The CRO market experienced considerable growth from 2010 to 2021 and is expected to continue growing through 2030. This growth is being driven by several key factors, including increased availability of research funding, government support for R&D, expansion of well-established healthcare sectors and healthcare expenditures, and a growing global patient population [5].

It is important to note that Africa is highly diverse, with substantial differences across countries and regions in terms of clinical research capacity, infrastructure, regulatory environments, and access to healthcare. These variations can influence the conduct and outcomes of clinical trials on the continent [6–9]. Although Africa has approximately 17.9% of the world’s population [10], only an estimated 2% of global clinical trials include at least one study site in Africa [11]. Currently, there is no publicly available data on CRO revenue capital in Africa and limited information exists regarding the number of CROs or their level of engagement in sub-Saharan Africa, a market space largely dominated by multinational CROs. As of 2025, South Africa is the leading hub for CROs in Africa, hosting approximately 60 CROs, a number significantly higher than other regions on the continent [12]. Moreover, to our knowledge, there are not many previous studies that show the identified challenges and opportunities of setting up and running CROs for the development of clinical trials in Africa. In the absence of official data, the importance of this industry segment is brought to light only by doing research.

The landscape of conducting clinical trials in Africa presents a range of perspectives, each with its own unique set of challenges and opportunities. As this landscape continues to evolve, CROs encounter several challenges that may impact their ability to conduct successful research [13].

The review presents results from various research articles on the opportunities and challenges of CROs in conducting clinical trials in Africa. It examines the association between these challenges and opportunities and the practices of CROs, as suggested by different authors.

## Methods

### Study identification and selection process

A comprehensive narrative review of published literature on CROs and clinical trials conducted in Africa was conducted. This included peer-reviewed journal articles including PubMed and Google Scholar using keywords such as: “challenges,” “opportunities,” “clinical trials,” “Contract research organizations,” “CROs,” and “Africa” in different combinations. The first 100 pages of Google were also reviewed to identify exemplary CROs for inclusion as an example. Other relevant sources of information, such as websites of CROs and organizations in the industry, were also searched. The search conducted on February 3, 2025, and publications available up to this date were considered.

## Results

### Challenges of clinical trial environment in Africa

#### Financial burden

The expenses involved in developing a drug, from initial research to getting it to market, can differ, but reports have suggested that these costs could exceed USD 2.5 billion [14]. In many low-income countries, conducting clinical trials may be considered a luxury. This is because the primary causes of death in these countries are diseases for which effective treatments exist but are not readily available due to insufficient funding [14]. For instance, in Ethiopia, the health sector received only 8.8% of the total national government spending in 2017/18 and 3.9% in 2023, below the 15% target set by the Abuja Declaration [15,16]. Developing countries get less than 10% of clinical trial funds even though they host 90% of the problems that affect the world’s population [29–30]. Funders and sponsors should understand that, with adequate funding, it is possible to conduct high-quality clinical trials in Africa [19]. One common challenge faced by CROs in Africa is the lack of funding opportunities for the growing number of trainees as they advance in their careers, as well as insufficient financial support for research projects [20]. Africa hosts a diverse range of CROs, including multinational branches, local private firms, and academic research units. This diversity helps reduce financial burdens by leveraging existing infrastructure, local expertise, and community trust to cut start-up, recruitment, and monitoring costs [21].

#### Limited human resource

Highly skilled personnel are essential for planning, initiating, and conducting clinical trials. Developing such human resources requires relatively stable, well-funded research and higher education institutions and robust science governance systems, often lacking in developing countries [22]. Graduates of medical schools and teaching hospitals in developing countries are often inadequately prepared to conduct clinical research [23]. Additionally, the increasing internationalization of the labor market is causing a continuous brain drain, as highly qualified personnel seek opportunities abroad [22]. Africa currently has 198 researchers per million people compared with 428 in Chile and loses some 20,000 professionals to high-income countries annually. Health professionals often struggle with understanding the importance of research techniques such as randomization, blinding, and tools like the visual analog scale [24]. In some oncological conditions, clinical trials are not feasible due to limited human resources, including a lack of pathologists and trained cancer surgeons, which in turn impacts the ability to provide proper cancer care [16]. All individuals involved in a clinical trial should receive proper training to ensure they comprehend the protocol and trial procedures. This is necessary to maintain consistency, adhere to the protocol, and uphold high standards. Certain sponsors, CROs, and regulatory agencies may require specific training or accredited trainers [25]. When sponsors want to outsource services depending on their needs, there should be enough trained people capable of investigating all services offered by CROs.

#### Infrastructure

In Africa, one of the challenges is the lack of a well-developed research infrastructure and a limited number of clinical trial units with administrative capacity [23]. According to a study done to assess medical laboratories in sub-Saharan Africa, there were only 380 laboratories qualified to meet international quality standards of which 91% were found in South Africa. Among 49 countries in the region, 37 did not have a single laboratory meeting international quality standards as per the evidence presented. Poor quality laboratory testing is a significant obstacle to providing quality health care in the region [17]. For various trials, CROs should have a complete setting,including adequate infrastructure and resources, that can able to do full scale trials, to attract sponsors and generate more complete results relevant to clinical practice [26].Strong CRO infrastructure in Africa is concentrated in South Africa, with its advanced trial sites, accredited laboratories, and established networks; Egypt, with modern hospitals and research facilities; Kenya, through well-developed vaccine and infectious disease research centers; and Nigeria, where expanding clinical sites are supported by academic and private investments [27].

#### The Ethical and regulatory system

An underdeveloped regulatory framework may result in the approval of poorly designed trials, which could pose significant risks to participants and communities that would be unethical and costly. In developing countries with fragile trust in health professionals and the healthcare system, unethical clinical trials can worsen negative attitudes toward health professionals, reduce health-seeking behavior, and foster dangerous mistrust in public health systems. Therefore, it is essential to create opportunities for meaningful communication among patients, researchers, and community physicians regarding clinical trials [26,28]. There should be an appealing environment for CROs to practice in and smooth regulatory mechanisms.

More than 70% of countries globally have weak national medicines regulatory systems [29]. In Africa, the number of African National Regulatory Authorities (NRAs) achieving Maturity Level 3 (ML3) status has increased from two in 2021 to eight at present: Egypt, South Africa, Tanzania, Nigeria, Ghana, Zimbabwe, Senegal, and Rwanda [30]. This remarkable progress highlights the importance of sustaining momentum; however, this figure remains below anticipated levels. Sponsors definitely prefer to choose sites in the countries where NRA with ML3 is working. The International Clinical Trial Registry Platform (ICTRP) data shows there is an increasing number of number of clinical trials in these countries [31].

These are different countries with different regulatory requirements. A combination of key local knowledge and close collaboration with the relevant authorities has enabled trial partners to overcome regulatory challenges. Key to managing this regulatory complexity is a combination of what approvals are required and how long it takes to receive approval. In Kenya, parties are required to submit Proforma Invoices (PFIs) and Certificates of Origin (COAs), which take 14 to 28 days to be granted. In Ghana, approval only takes 8 to 10 days, and additional permit fees and a valid license of importer must also be submitted [32].

Generally different perspectives should be well considered in conducting clinical trials in developing countries as CROs where easily preventable communicable diseases are of major concern and account for the highest death toll. The financial and human resources, infrastructure, ethical and regulatory contexts, cultural appropriateness, and living conditions of host communities are triggering the question of whether clinical trials and CROs are important in developing countries.

### Opportunities of clinical trial environment in Africa

Fast growing population, genetic diversity, epidemiological transition, a rising middle class, and rapid economic growth are some of the opportunities for conducting clinical trials in Africa. Low access to quality healthcare can be both opportunity and challenge. It can attract more patients to be enrolled for the “free and high-quality treatment.” At the same time, low access can make it difficult to have the correct diagnosis.

#### Large number of populations

Africa is the second-largest and second-most-populous continent with an estimated population of approximately 1.55 billion people as of mid-2025, representing about 18.8% of the total world population [33].Despite the large number of population, the continent is underrepresented in clinical trial [34]. This large number of populations has special benefits for choosing Africa to conduct clinical trials. The large proportion of the population with limited prior research participation allows CROs to build extensive volunteer pools, which presents a significant opportunity for rapid, large-scale participant recruitment for sponsor-related activities.

#### Genetic diversity

African populations have the highest levels of genetic variation among all humans and host more than 3000 ethnic groups [35,36]. For example, Ethiopia’s population is also highly diverse, containing over 80 ethnic groups [37]. This incredible number of ethnic groups shows a diversified genetic makeup among the whole population. Genetic analyses show that various ethnic groups have variable results in different treatments. For example, some interventions shown to be efficacious in high-income countries are not similarly effective when used in other contexts [7]. These genetic factors can modulate responses to many drugs, as well as local environmental factors that can influence clinical expression of an underlying disease [38]. This is an excellent opportunity for CROs to carry out clinical trials in Africa.

#### Diversity of disease

Most Africans are suffering from a double disease burden [39], comprising communicable and non-communicable diseases. This means that there is an abundance of the patient population representing various disease areas, which further indicates an opportunity for rigorous scientific investigation and breakthroughs.

While infectious diseases may command the most attention, the continent also experiences a significant burden of non-communicable diseases. A World Bank report indicates that by 2030, non-communicable diseases are expected to cause more deaths in Africa than communicable diseases [40]. This is largely due to the increasing number of deaths from cardiovascular disease among people under 70 years of age in sub-Saharan Africa [41]. Cancer now kills more Africans than malaria, while conditions like type II diabetes and cardiovascular disease are on the rise. Gynecologic malignancies such as breast, cervical, and uterine cancers represent the first, third, and fifth most common cancers in women globally [42]. According to a study conducted in sub-Saharan Africa, gynecological cancers account for approximately one-third of all female cancers. Among these, cervical cancer is the most prevalent, constituting 81.6% of all female cancers [43]. Clinical trials play a crucial role globally in lessening the burden of diseases by contributing to the creation of safe and effective new treatments and vaccines [25]. CROs can take advantage of these opportunities to alleviate such disease loads.

### Lower operating cost

The operational costs in many African countries are competitive compared with more saturated markets, making Africa an appealing destination for pharmaceutical companies and CROs [44].The cost of running a trial is significantly cheaper in developing countries than in developed countries. This is largely due to lower salaries, overhead costs, and shorter participant enrollment times [45]. Although precise percentage data specific to Africa are limited, evidence from clinical trials conducted in emerging markets, including those in Africa, indicates substantial overall cost reductions, ranging from approximately 35% to 60%, primarily driven by lower labor costs, more efficient patient recruitment, and shorter study timelines compared to trials conducted in the United States or Europe [46].

According to reports from pharmaceutical sponsors, a second-tier center in the USA charges more than ten times the amount that a top-tier academic medical center in India charges per case report [47].

Other studies estimated that a trial in China or India could cost between a third to half of what it would cost in the USA [48]. Recruitment of trial participants is easier than in the developed world; large outcome trials that require the enrollment of thousands of patients could make huge savings for the sponsor if the trial is conducted outside of developed countries [49]. Subject recruitment alone accounts for approximately 23% of total trial costs, and 87% of US trials fail to meet their recruitment and enrollment milestones [50].

#### Positive economic outlook

The World Bank forecast notable economic growth in different sectors in sub-Saharan Africa. In 2024, the growth rate in sub-Saharan Africa is expected to increase to 3.5%, and then average around 3.9% in the years 2025–2026. This is anticipated as inflation decreases and there are improvements in private consumption and investment [51]. Security threats have subsided in several countries, and infrastructure investments to sustain growth are improving. A growing emphasis from both government and private sectors is being placed on luring biomedical research initiatives. This shift toward increased investment in science and technology in Africa holds promise for advancing medical research in the region [52].

#### Increasing global health players

Africa has become a key part of the global health landscape. Increasingly, there have been many clinical trial partnerships between Africa’s academic research universities and those from the West. Expatriate biomedical scientists are welcomed to teaching hospitals and public research institutions in Africa to undertake research involving human subjects. Teaching and referral hospitals and African physicians are keen to participate in clinical trials and are hungry for research opportunities and grants for their institutions. Patient and researcher priorities in Africa are often aligned with the needs of pharmaceutical sponsors. The abundance of well-trained English-speaking researchers and the availability of English source documents make the African continent attractive for conducting clinical trials [52].

#### Research centers

Conducting clinical research in low- and middle-income countries (LMICs) contributes significantly to capacity building in terms of training and infrastructure, which enhances the overall public health benefits of the country [53]. The benefits include staff acquiring experience in conducting research that meets global standards and receiving training in International Council for Harmonization-Good Clinical Practice (ICH/GCP), clinical, laboratory, and data management procedures, as well as the utilization of new equipment and vaccine safety assessment.

The research teams gain national and international exposure, and there are enhanced opportunities for future research funding at the center [54]. CROs bring these benefits to patients, healthcare workers, and the community before, during, and after research.

As of January 16, 2025, ClinicalTrials.gov showed 55% of registered studies as having a “non-USA-only” location. 30% were USA-only registered studies and more than 65% of them were recruited outside of the USA [55]. This shows that many registered studies in USA are physically conducted in countries like developing nations.

### Best practices from successful CROs operating in Africa

A few accredited CROs in the region can conduct local clinical trials [56]. We have put some successful CROs operating in different African regions.

Oximio operates its own hub in Kenya, which significantly streamlines infrastructure challenges for the CRO. This hub has proven instrumental in accelerating distribution processes across Africa, particularly for multicenter trials. With a dedicated depot in Kenya, Oximio is able to rapidly dispatch supplies to other countries on the continent, even at short notice [32]. In addition to speeding up distribution processes across Africa, an established, local network between trial partners and the target region will enable significant cost and administration savings to be delivered through the clinical trials process.

P95 is a full-service CRO specializing in vaccine development and infectious diseases. They operate across more than 200 sites, with local hubs in South Africa, Ghana, Kenya, and Morocco, allowing them to deploy expertise quickly and efficiently. They have conducted over 500 clinical trials across 20+ African countries, involving up to 3,000 participants [57].

ICON is supporting clinical trial execution in West Africa during the Ebola outbreak, including a long-term survivor study. Recognizing the urgent need, they are assessing whether convalescent plasma from Ebola survivors could effectively treat Ebola virus disease [58]. This highlights how CROs actively engage in critical outbreak situations to support global health initiatives.

MCT-CRO is the only CRO capable of covering French, English, and Portuguese-speaking regions across sub-Saharan Africa. Operating in over 20 countries, this multilingual capability provides a unique strategic advantage, enhancing the efficiency of clinical trial management. With MCT-CRO, clients benefit from reduced travel time, improved site management, and more profound local knowledge, including expertise in regulatory processes, identifying top sites, working with local vendors, and navigating healthcare system organizations [59].

Some CROs, like ACRO, develop a toolkit that facilitates the implementation of decentralized clinical trials (DCT). For example, their DCT toolkit provides a detailed analysis of design and execution considerations in undertaking a DCT, as well as a risk assessment tool and maps that illustrate how data is gathered, transmitted, accessed, and protected in DCTs, an important consideration for regulators, patients, and others [60].

Another topic on which the CROs and technology companies will lead in the future is the use of real-world data/real-world evidence (RWD/RWE), which will not only provide more fulsome data for drug development but ultimately could help to begin bridging the gap between clinical research and clinical care [61]. Emerging new technologies could reshape clinical trials over the long term. The adoption of technology is expected to become increasingly widespread over time. While CROs are only beginning to explore innovative technologies such as artificial intelligence (AI) and machine learning, the growth of technology may lead to a shift in the CRO model from being primarily labor-driven to being more technology-driven [62].

### Emerging trends and future outlook for CROs in Africa

#### Decentralized clinical trials

In sub-Saharan Africa, the transition to DCTs is at an early stage. With the outset and rapid spread of SARS-CoV-2, DCTs were expeditiously initiated in some research institutions and CROs in sub-Saharan Africa to allow continuity of ongoing clinical trials despite the unfolding pandemic [21].

However, Africa is several years ahead of the curve in terms of remote trial adoption due to a combination of high mobile phone usage, how widely disbursed the population is, and, in particular, previous experience in remote trials conducted during the Ebola crisis in 2014. As a result, Africa is well placed to benefit from such approaches as they continue to grow in prominence.

#### Conducting clinical trials in public health emergencies

The health systems in Africa have been under enormous pressure - a pressure that coexists with and is aggravated by public health emergencies and other disasters and challenges in Africa, such as natural disasters and socioeconomic, political, and conflict situations [63]. Countries in the World Health Organization (WHO) African region experience over 100 health emergencies annually, which weakens already fragile health systems [64]. Recently Africa Center for Diseases Control (CDC) officially declared theongoing Mpox outbreak a Public Health Emergency of Continental Security (PHECS), marking the first such declaration by the agency since its inception in 2017 [65]. Doing clinical research is a must in public health emergencies, and there were experiences in COVID-19 and Ebola. The clinical trials during the 2014–2015 Ebola epidemic were conducted in an atmosphere and on a timeline entirely different from most clinical trials [66]. CROs and other stakeholders played a critical role in accelerating data collection, informing clinical management of patients, and assessing the safety, efficacy, and effectiveness of therapeutics and vaccines. Additionally, they contributed to enhancing outbreak control efforts and improving response strategies.

#### Conceptual framework

**Figure 1.**
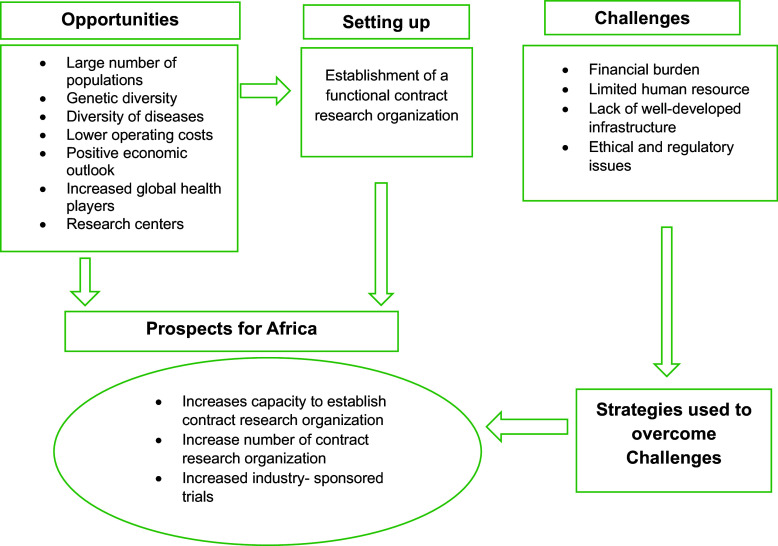
Conceptual framework the researcher developed from the literature, depicting the opportunities, setup, challenges faced, strategies, and prospects of contract research organizations. This figure presents the conceptual framework developed by the researcher from the literature, illustrating the various components related to Contract Research Organizations (CROs). The framework depicts key aspects such as opportunities, setup, challenges faced, strategies, and prospects in the context of CROs. Arrows represent the relationships between these components, and boxes represent each area of focus.

A conceptual framework based on the setup, opportunities, challenges faced, possible strategies, and prospects of CROs is depicted in Figure 1.

This framework synthesizes existing literature to demonstrate the intricate interrelationships among these variables. It provides a comprehensive visual representation, showing how various opportunities have facilitated the establishment of CROs. Additionally, it highlights the challenges that CROs encounter, which may hinder progress if not addressed effectively. Crucially, the framework also outlines the strategic responses employed by CROs to overcome these challenges, ultimately contributing to the growth and development of the industry.

### Limitations

In Africa, many CROs are international and operate in multiple countries [67]. Unfortunately, local CROs often lack websites and online services, making it difficult for them to communicate the challenges they face [68]. Furthermore, this study does not provide standardized metrics to measure the success of these organizations; instead, it relies on various data from different sources with varying scope and quality, which may limit the ability to draw definitive conclusions about their overall contributions. Finally, as a narrative review, only selected keywords were used, so some relevant studies may not have been captured.

## Conclusions

Conducting clinical trials in Africa presents a unique set of challenges and opportunities. These challenges and opportunities are interconnected and hinder the growth of CROs and African clinical trials. While obstacles such as limited infrastructure, financial constraints, insufficient skilled human resources, and regulatory complexities continue to hinder the successful implementation of clinical trials, the region’s potential remains significant. Africa’s large, diverse population, genetic variations, and the growing burden of both communicable and non-communicable diseases offer a wealth of opportunities for impactful clinical research. Furthermore, the continent’s lower operational costs, improving economic conditions, and increasing involvement in global health initiatives position it as an emerging hub for clinical trials. To unlock this potential, it is essential to address the systemic challenges, enhance capacity building for CROs, keep the sites prepared for the next studies, a sustainability challenge, and strengthen regulatory frameworks. There is a pressing need for continued research to understand better the specific challenges faced by CROs and develop effective strategies to navigate these issues. By capitalizing on the region’s strengths and addressing its challenges, Africa can become a more attractive and viable destination for conducting clinical trials, ultimately contributing to advancing global healthcare.
